# Edible Bird’s nest extract as a chondro-protective agent for human chondrocytes isolated from osteoarthritic knee: in vitro study

**DOI:** 10.1186/1472-6882-13-19

**Published:** 2013-01-22

**Authors:** Kien-Hui Chua, Ting-Hun Lee, Kamini Nagandran, Nor Hamdan Md Yahaya, Chew-Tin Lee, Eddie Tan Ti Tjih, Ramlan Abdul Aziz

**Affiliations:** 1 Department of Physiology, Faculty of Medicine, Universiti Kebangsaan Malaysia, Jalan Raja Muda Abdul Aziz, Kuala Lumpur, 50300, Malaysia; 2 Institute of Bioproduct Development, Universiti Teknologi Malaysia, Skudai, Johor 81310, Malaysia; 3 Department of Orthopaedic and Traumatology, Faculty of Medicine, Universiti Kebangsaan Malaysia, Jalan Yaacob Latif Bandar Tun Razak, Kuala Lumpur, Cheras, 56000, Malaysia; 4 Faculty of Chemical Engineering, Universiti Teknologi Malaysia, Skudai, Johor, 81310, Malaysia; 5 Food Technology Department, Faculty of Applied Sciences, Universiti Teknologi MARA, Selangor, Shah Alam, 40450, Malaysia

## Abstract

**Background:**

Osteoarthritis (OA) is a degenerative joint disease that results in the destruction of cartilage. Edible Bird’s Nest (EBN) extract contains important components, which can reduce the progression of osteoarthritis and helps in the regeneration of the cartilage. The present study aimed to investigate the effect of EBN extract on the catabolic and anabolic activities of the human articular chondrocytes (HACs) isolated from the knee joint of patients with OA.

**Methods:**

A single batch of EBN extract was prepared with hot-water extraction and coded as HMG. HACs were isolated from the knee joint cartilage removed during surgery. The optimum concentration of HMG for HAC cultures was determined using MTT assay. The effect of HMG on the catabolic and anabolic genes’ expression in HACs was measured by real-time PCR. The total amount of prostaglandin E_2_ (PGE_2_) production was determined by ELISA method, and the total sulphated glycosaminoglycan (GAGs) production was quantified by 1,9-dimethylmethylene blue (DMMB) assay.

**Results:**

MTT assay showed 0.50% - 1.00% HMG supplementation promoted HACs proliferation. HMG supplementation was able to reduce the catabolic genes’ expression in cultured HACs such as matrix metalloproteinases (MMP1 & MMP3), Interleukin 1, 6 and 8 (IL-1, IL-6 & IL-8), cyclooxygenase-2 (COX-2) and inducible nitric oxide synthase (iNOS). Prostaglandin E_2_ (PGE_2_) production was significantly reduced in HAC cultures supplemented with HMG. With regard to anabolic activity assessment, type II collagen, Aggrecan and SOX-9 gene expression as well as sGAG production was increased in the HMG supplemented groups.

**Conclusion:**

Edible Bird’s Nest extract coded as HMG demonstrated chondro-protection ability on human articular chondrocytes in vitro. It reduced catabolic activities and increased cartilage extracellular matrix synthesis. It is concluded that HMG is a potential agent in the treatment of osteoarthritis.

## Background

Osteoarthritis (OA) results in progressive degeneration of the articular cartilage. OA is caused by the increase in the production of matrix metalloproteinases (MMPs), pro-inflammatory cytokines and catabolic mediators which destroy the cartilage matrix [1]. Usually, there is a balance in the anabolic and catabolic activities in the articular chondrocytes which are involved in the remodelling of the extracellular matrix (ECM). Disturbance in the normal balance triggers the pathological changes in OA and this causes an increase in inflammatory factors and cytokine gene expressions [2]. Pro-inflammatory cytokines such as Interleukin-1β, 6 and 8 (IL-1β, IL-6 and IL-8) initiate the development of OA by increasing the number of inflammatory cells in the synovial tissue thereby increasing the amount of MMPs and inhibiting the production of proteoglycans [3,4]. Subsequently, the release of the catabolic mediators such as Prostaglandins E_2_ (PGE_2_), cyclooxygenase-2 (COX-2) and inducible nitric oxide synthase (iNOS), contribute to the catabolic consequences of OA [5].

Normal articular cartilage is composed of highly organized proteoglycan and collagen network. Aggrecan along with hyaluronic acid creates huge proteoglycan aggregates, and forms negatively charged glycosaminoglycan (GAG). The structure rigidity of cartilage is conferred mainly by type II collagen. Type II collagen is destroyed in cartilage injury, and it is replaced by type I collagen which possess inferior functions [6]. The degradation of both aggrecan and type II collagen contribute to the progression of OA [7,8].

One of the recent symptomatic treatments employed for OA involves the use of non-steroidal anti-inflammatory drugs (NSAIDs) to block cyclooxygenase (COX) [9]. NSAIDs cause an increase in damage to the cartilage. On the other hand, usage of COX-2 specific class of NSAIDs is known to cause cardiovascular and heart diseases [10]. Thus, there is an urgent need to identify and develop new approaches to treat or inhibit the progression of OA.

Edible Bird’s Nest (EBN) was a significant item in the cuisine and pharmacy of the Emperors of China during the 16^th^ century [11]. EBN has been known for its beneficial effect in promoting health in the Chinese for thousands of years [12]. In Traditional Chinese Medicine (TCM), EBN is believed to promote the wellbeing of multiple organs and body systems [13,14]. Previous study showed an avian epidermal growth factor (EGF) extracted from EBN improved the immune function and cell proliferation [15]. Hiroki Nakagawa *et al.,* 2007 [16] found that EBN is rich in proteoglycans containing non-sulfated chondroitin glycosaminoglycan (GAGs) which shares similar properties like the matrix in the cartilage. The proteoglycans isolated contained 83% carbohydrates, of which 79% were GalNAc and GlcUA (D-glucuronic acid) in an equimolar ratio. Recently, hot-water extraction of EBN showed the ability to promote cornea cells proliferation and cornea wound healing [17]. To date, all the previous studies demonstrated the beneficial effect of EBN extract prepared by water extraction. Hence, it is hypothesized the water extraction of EBN may prevent human articular chondrocytes (HACs) lose its functions.

Since EBN is produced from swiftlet’s saliva, extraction method using organic solvent such as methanol, chloroform and DMSO, which target the non-water soluble active compound, may not be practical. The EBN extract prepared by water extraction mainly contains polysaccharide and glycoprotein, which can easily precipitate through freezing and thawing processes. Thus, we prepared the EBN water extract in a single batch and stored at 4°C in order to maintain the uniformity and quality of the extract for the experiment. The present study aimed to measure the effects of EBN extract on the anabolic and catabolic activities of HACs.

## Methods

### Edible Bird’s nest extract

Edible Bird’s Nest (EBN) extract coded as HMG was obtained from Chemical Engineering Pilot Plant (CEPP), Universiti Teknologi Malaysia (UTM) using an in-house developed method adapted from Oda *et al*., 1998 [18]. The EBN was collected from a bird’s house in Batu Pahat, Johor. Briefly, EBN was grounded in a mortar and pastel; sieved through 0.4 mm wire mesh to separate feathers and other impurities. The grounded EBN then underwent hot-water extraction at 80°C. The liquid was then centrifuged to remove non-dissolved constituents. The EBN extract was stored at 4°C until used.

### Human articular chondrocytes (HACs) isolation and culture

Prior ethical approval was obtained from the Research and Ethical Committee of Faculty of Medicine, Universiti Kebangsaan Malaysia (UKM project code: 06-01-06-SF0257). Cartilage was obtained from six osteoarthritic patients aged between 55–70 years old, who gave their consent during total knee replacement surgery (TKR). All patients had OA knee with lesion scored by International Cartilage Repair Society (ICRS) of grade 4. The cartilage was harvested from the non-weight bearing area of intercondylar notch distal femur. The specimen needed to have a minimum of 300 mg remnant good cartilage, and the isolated HACs expressed collagen type II mRNA with ± 20% differences from the average. Each harvested specimen was placed in normal saline and transported to Biotechnology Laboratory in the Department of Physiology, Faculty of Medicine, UKM within 24 hours. Each specimen was processed individually to isolate the HACs. The cartilage was separated from the bone, washed with phosphate buffered saline (PBS; pH 7.2, Gibco, Grand Island, NY), minced into small pieces and digested with 0.6% type II collagenase (Worthington Biochemical Corporation, New Jersey) in an orbital incubator at 37°C for 6–8 hours. The cell suspension was then centrifuged at 600xg for 10 min to pellet the HACs. The isolated HACs were then cultured in growth medium which consisted of Ham’s F-12:Dulbecco’s Modified Eagle’s Medium (Gibco), 10% foetal bovine serum (FBS; Gibco), 1% of antibiotic and antimycotic (Gibco), 1% of 50 μg/ml ascorbic acid (Sigma, St. Louis, USA) in T25 culture flasks (Nunc, Denmark). All cultures were maintained at 5% CO_2_ in a temperature of 37°C. Upon 80% confluence, the HACs (Passage 0) were trypsinized using 0.125% trypsin-EDTA (Gibco) and sub-culture (passage 1) into 6-well plates (Nunc) at the 10,000cells/cm^2^ seeding density. HACs were incubated overnight in growth medium to allow cell attachment before changed to culture medium without FBS and ascorbic acid but supplemented with HMG at various concentrations ranging from 0.00% (control) to 3.00%, for seven days.

### MTT assay

The proliferation rate of each cultured HACs in different concentration of HMG was determined by MTT Assay using commercial kit (Sigma, USA) following seven days of culture. The assay was started by adding 2 ml of medium containing 0.5 mg/ml MTT into each culture wells and incubated for 4 hours in 5% CO_2_ incubator at 37°C. Consequently, the formed formazan crystal was dissolved by adding 2 ml of MTT Solubilization Solution. The solution was then transferred into 1.5 ml cuvette and the absorbance at 570 nm was taken three times. Two HMG concentrations that gave the highest cell proliferation were then used for the subsequent experiments.

### Total RNA extraction

Total RNA from cultured HACs either in HMG supplemented or control group was extracted using TRI REAGENT (Molecular Research Center, Cincinnati, USA) according to the manufacturer’s protocol. Briefly, chloroform was added into the TRI REAGENT homogenate to separate the colourless aqueous contained total RNA. Next, the total RNA was precipitated from the aqueous by mixing with isopropanol and 10 μl of Polyacryl carrier (Molecular Research Center). The precipitated RNA was washed with 75% ethanol and air-dried before being dissolved in water. The yield and the purity of the total RNA was determined by spectrophotometer (Bio-Rad, Hercules, USA).

### CDNA synthesis

SuperScript™ III First-Strand Synthesis SuperMix (Invitrogen, Carlsbad, USA) was used to synthesis cDNA from extracted total RNA according to the manufacturer’s protocol. The reverse transcription was carried out at 50.0°C for 30 minutes and the cDNA was stored at −20°C until use.

### Quantitative polymerase chain reaction (Q PCR)

Primers used for quantitative PCR were designed based on the sequences published in GenBank using Primer-3 software. The primers sequenced were listed (Table 1). Quantitative PCR was performed at 94°C (pre-denaturation step), 2 min; 40 cycles of denaturation at 94°C, 10 sec; amplification at 61°C, 30 sec and followed by melting curve analysis to verify the specificity of the PCR products. The specificity of PCR product was also examined by agarose gel electrophoresis. Glycerylaldehyde-3-phosphate dehydrogenase (GAPDH) was used as the reference gene for the data normalization.

**Table 1:** Primers sequence of genes for quantitative PCR

**Gene Name**	**GenBank Accession Number**	**Primer Sequence 5′-3′**	**PCR product size (bp)**
GAPDH	NM_002046	F: 5′-tcc ctg agc tga acg gga ag-3′	217
		R: 5′-gga gga gtg ggt gtc gct gt-3′	
COL I	NM_000088	F: 5′-agg gct cca acg aga tcg aga-3′	222
		R: 5′-tac agg aag cag aca ggg cca-3′	
COL II	NM_001844	F: 5′-cta tct gga cga agc agc tgg ca-3′	209
		R: 5′-atg ggt gca atg tca atg atgg-3′	
ACP	NM_001135	F: 5′-gcg gag gaa gtc ggt gaa ga-3′	183
		R: 5′-ccc tct cgc ttc agg tca gc-3′	
SOX-9	NM_000346	F: 5′-cac tgt tac cgc cac ttc cc-3′	236
		R: 5′-acc agc gga agt ccc ctt cg-3′	
IL-1	NM_000576	F: 5′-gga caa gct gag gaa gat gc-3′	120
		R: 5′-tcg tta tcc cat gtg tcg aa-3′	
IL-6	NM_000600	F: 5′-tac ccc cag gag aag att cc-3′	175
		R: 5′-ttt tct gaa agt gcc tct tt-3′	
IL-8	NM_000584	F: 5′-gtg cag ttt tgc caa gga gt-3′	196
		R: 5′-ctc tgc acc cag ttt tcc tt-3′	
MMP1	NM_002421	F: 5′-agg gtt gaa aag cat gag ca-3′	111
		R: 5′-ctg gtt gaa aag cat gag ca-3′	
MMP3	NM_002422	F: 5′-tgc ttt gtc ctt tga tgc tg-3′	135
		R: 5′-gga aga gat ggc caa aat ga-3′	
MMP13	NM_002427	F: 5′-ggt ctt gac cac tcc aag gac-3′	221
		R: 5′-ctc ctc gga gac tgg taa tgg-3′	
iNOS	NM_000625	F: 5′-aca agc cta ccc ctc cag at-3′	158
		R: 5′-tcc cgt cag ttg gta ggt tc-3′	
COX-2	NM_000963	F: 5′-tga gca tct acg gtt tgc tg-3′	158
		R: 5′-tgc ttg tct gga aca act gc-3′	

### Prostaglandin E2 (PGE_2_) quantification

The culture medium from the cultured HACs at day seven was collected and kept frozen until the concentration of Prostaglandins E_2_ (PGE_2_) was measured with Parameter™ PGE_2_ Immunoassay Kit (R&D Systems Inc, USA). The sample preparation and reaction protocol was following the manufacturer’s instruction. The absorbance at 450 nm was acquired three times. A standard curve was plotted, and the amount of PGE2 released in each sample was determined from the standard curve.

### Sulphated glycosaminoglycan (sGAG) quantification

The amount of sGAG in each group was measured by 1,9-dimethylmethylene blue (DMMB) assay. Chondroitin Sulfate S (Sigma) was used as the control to plot the standard curve. The sGAG concentration in the sample was normalised against the DNA content in the respective specimens.

## Results

### HACs proliferation with HMG supplementation

**Figure 1:**
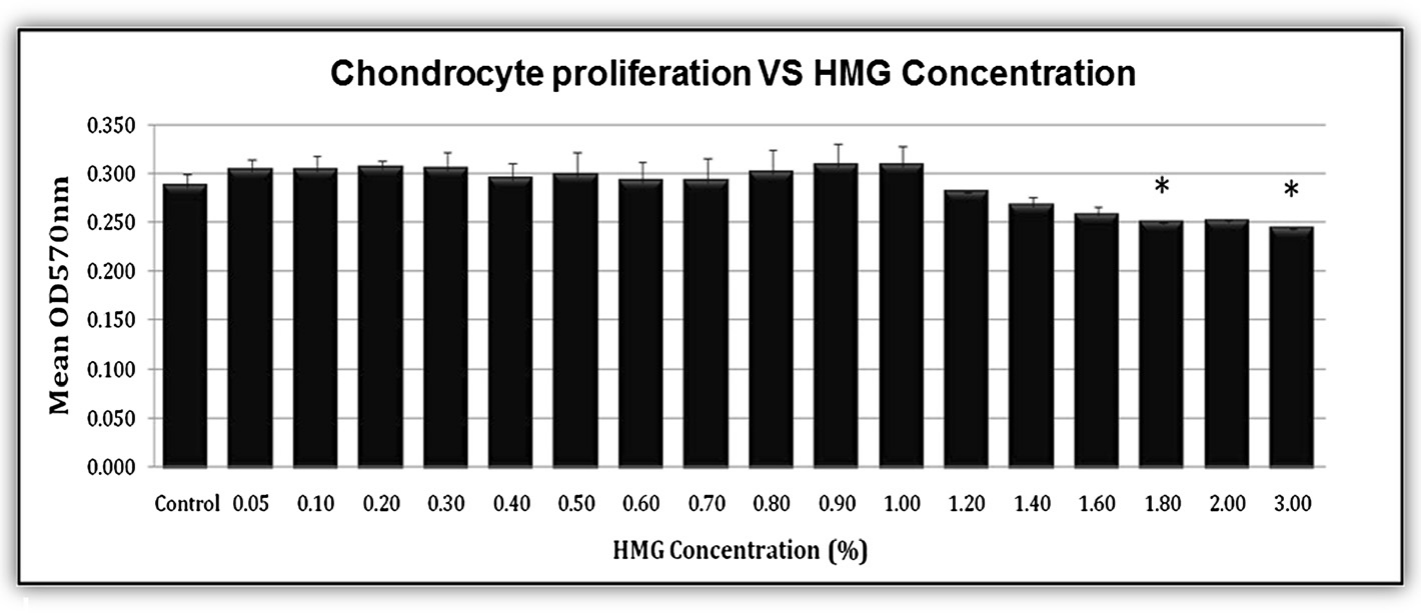
**Human articular chondrocytes cultured in various concentration of HMG in the culture medium.** The values were expressed as mean ± SEM, n=6. *Significant lower proliferation compared to the control group (P<0.05%).

The results showed HMG supplementation at low concentration (0.05% - 1.00%) promoted the proliferation of HACs (Figure 1). However, HACs proliferation was slower than the control group when HMG concentration was higher than 1.00%. HACs recorded significant lower proliferation in cultures supplemented with HMG at 1.80% and 3.00% concentration. Subsequently, HMG at concentration of 0.50% and 1.00% were chosen for the following tests.

### Morphological features of HACs in the culture

**Figure 2:**
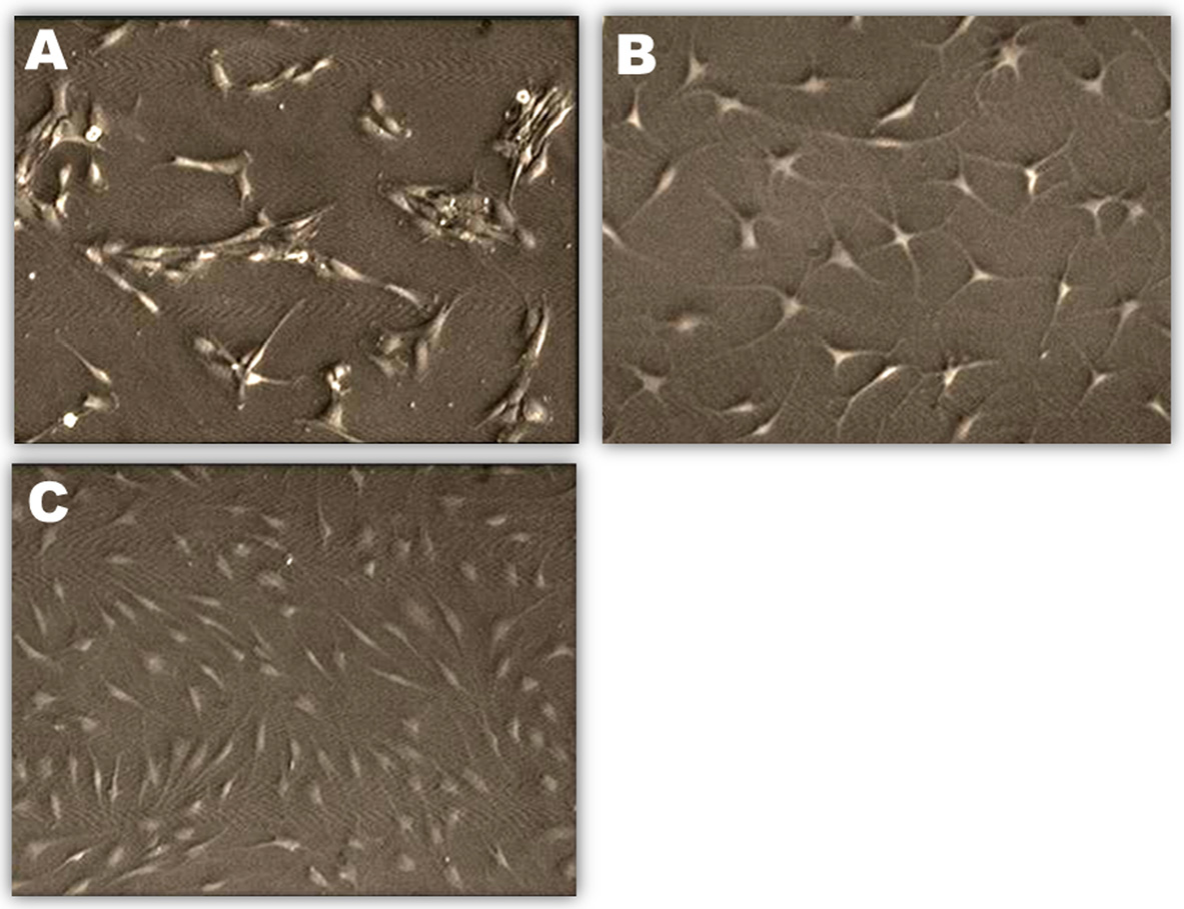
**Morphological features of cultured human articular chondrocytes (HACs). A**) Freshly isolated HACs cultured in medium nourished with 10% FBS, **B**) HACs cultured in medium without FBS (control), **C**) HACs cultured in medium supplemented with 1.00% HMG. Magnification 100×.

The morphological feature of HACs cultured in various HMG concentrations was captured under an inverted microscope (Figure 2). HACs cultured at P0 in the medium supplemented with 10% FBS appeared in polygonal shape (Figure 2A). When HACs were cultured in the medium without FBS, it lost its polygonal shape and changed to dendrite-like features (Figure 2B). HACs cultured in medium supplemented with HMG recovered partially of the polygonal feature with some cells appearing as fibroblastic (Figure 2C).

### HMG effects on the mRNA expression of HACs

**Figure 3:**
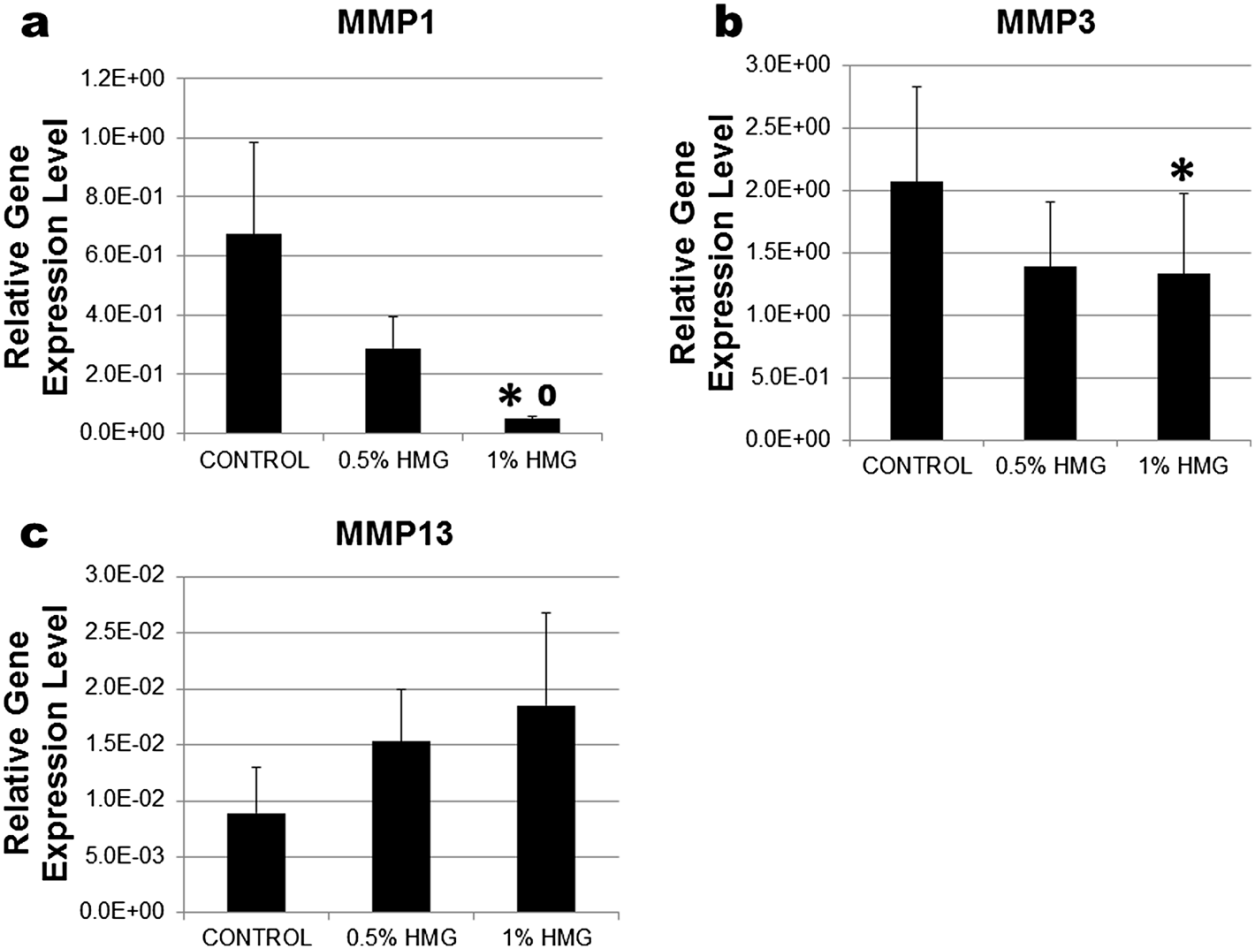
**Relative gene expression level of matrix metalloproteinase in HACs cultured in medium supplemented with HMG. 3a) MMP1, 3b) MMP3 and 3c) MMP13.** The values were expressed as mean ± SEM, n=6. *Significant different compared to the control group (P<0.05%). ^0^Significant different between HMG concentration (P < 0.05%).

HMG supplementation was able to reduce HACs expression on MMP1 and MMP3 mRNA in a dose-dependent manner (Figure 3a and 3b). HMG concentration at 1.00% significantly reduced the MMP1 expression by −13.27fold compared to the control group (P=0.010) and −5.67fold compared to the 0.5% HMG group (P=0.016). HMG concentration at 1.00% significantly decreased MMP3 expression by −1.56fold (P=0.047) compared to the control group. However, HMG caused no significant increment in the MMP13 expression in HACs compared to the control group (Figure 3c).

**Figure 4:**
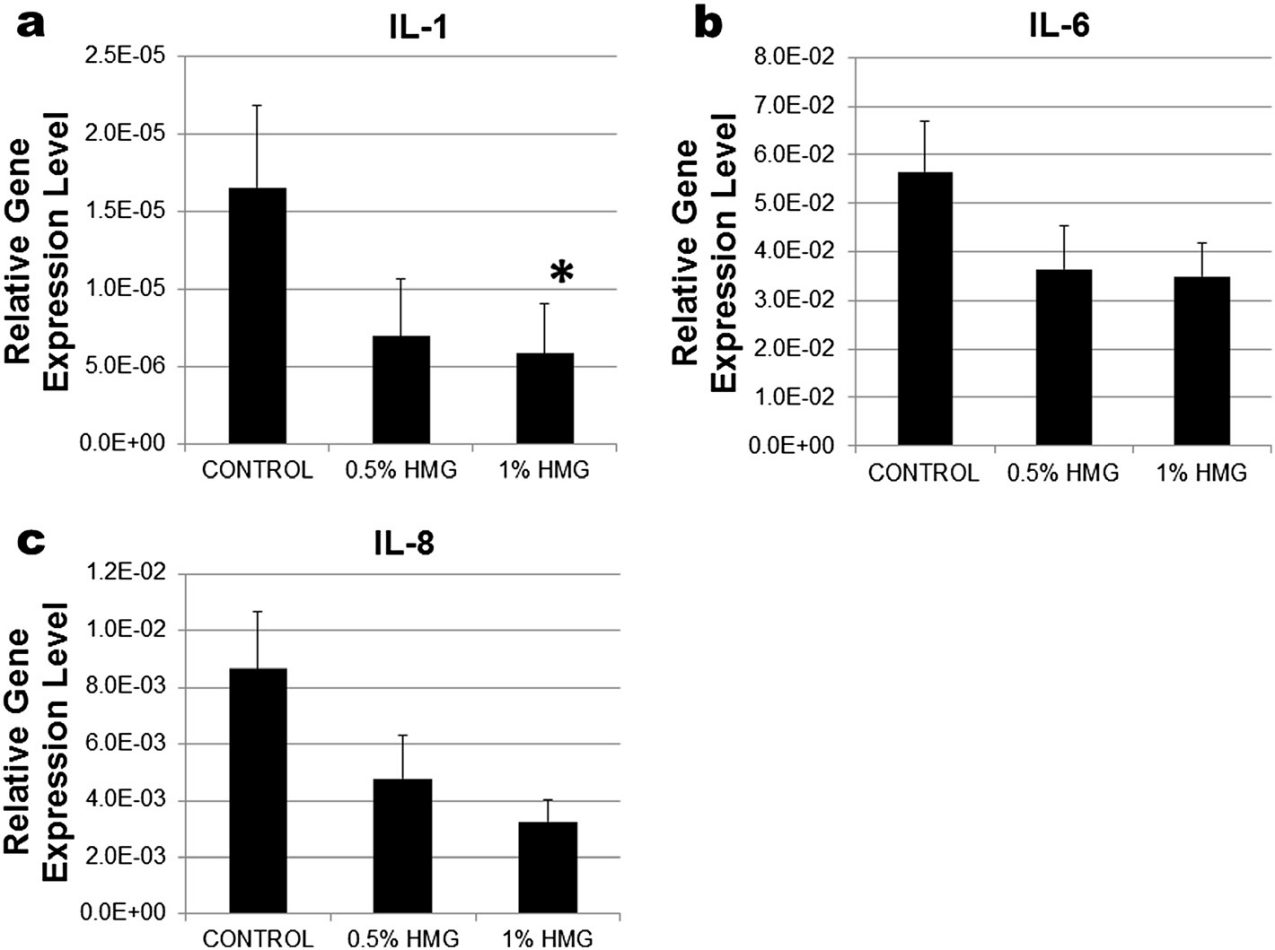
**Relative gene expression level of pro-inflammatory markers in HACs cultured in medium supplemented with HMG. 4a) IL-1, 4b) IL-6 and 4c) IL-8.** The values were expressed as mean ± SEM, n=6. *Significant different compare to the control group (P < 0.05%).

**Figure 5:**
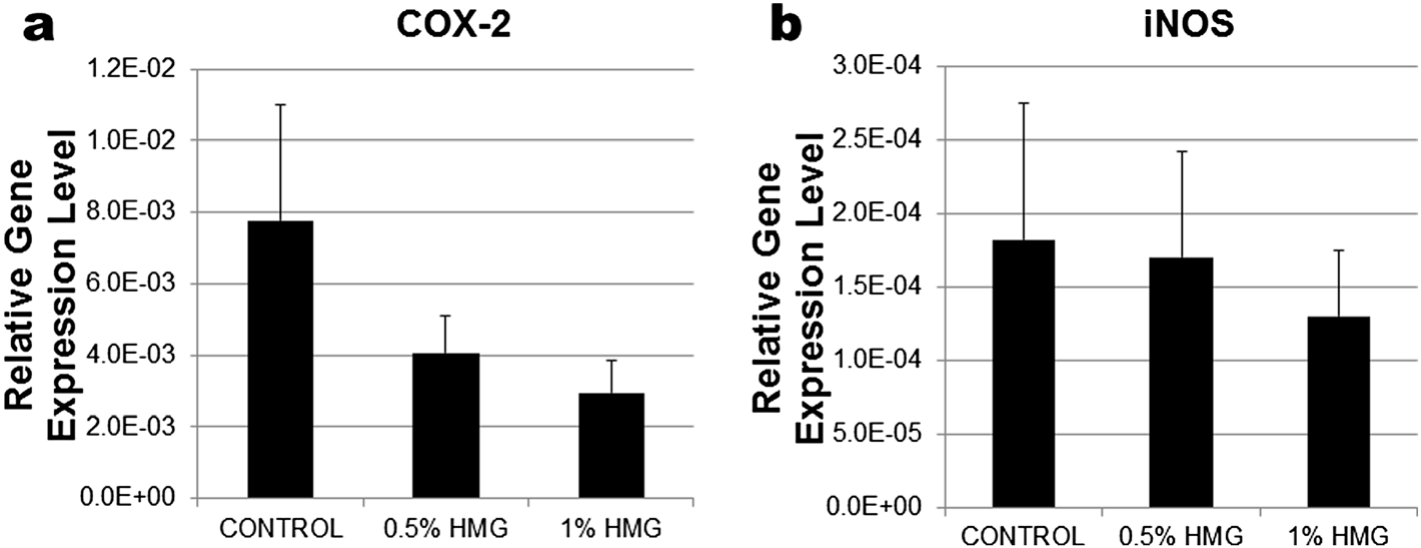
**Relative gene expression level of 5a) COX-2 and 5b) iNOS in HACs cultured in medium supplemented with HMG.** The values were expressed as mean ± SEM, n=6.

HMG also caused down-regulation of IL-1, IL-6 and IL-8 mRNA expression in HACs (Figure 4). However, only the pro-inflammatory marker, IL-1 expression was significantly decreased by 1.00% HMG supplementation (−2.38fold, P=0.025; Figure 4a). Both the catabolic mediators; COX-2 and iNOS mRNA expression in HACs reduced with the HMG supplementation (Figure 5). HMG at 1.00% concentration reduced the COX-2 expression by −2.64fold and iNOS expression by −1.4fold in cultured HACs. However, the reduction was not significant compared to the control group.

**Figure 6:**
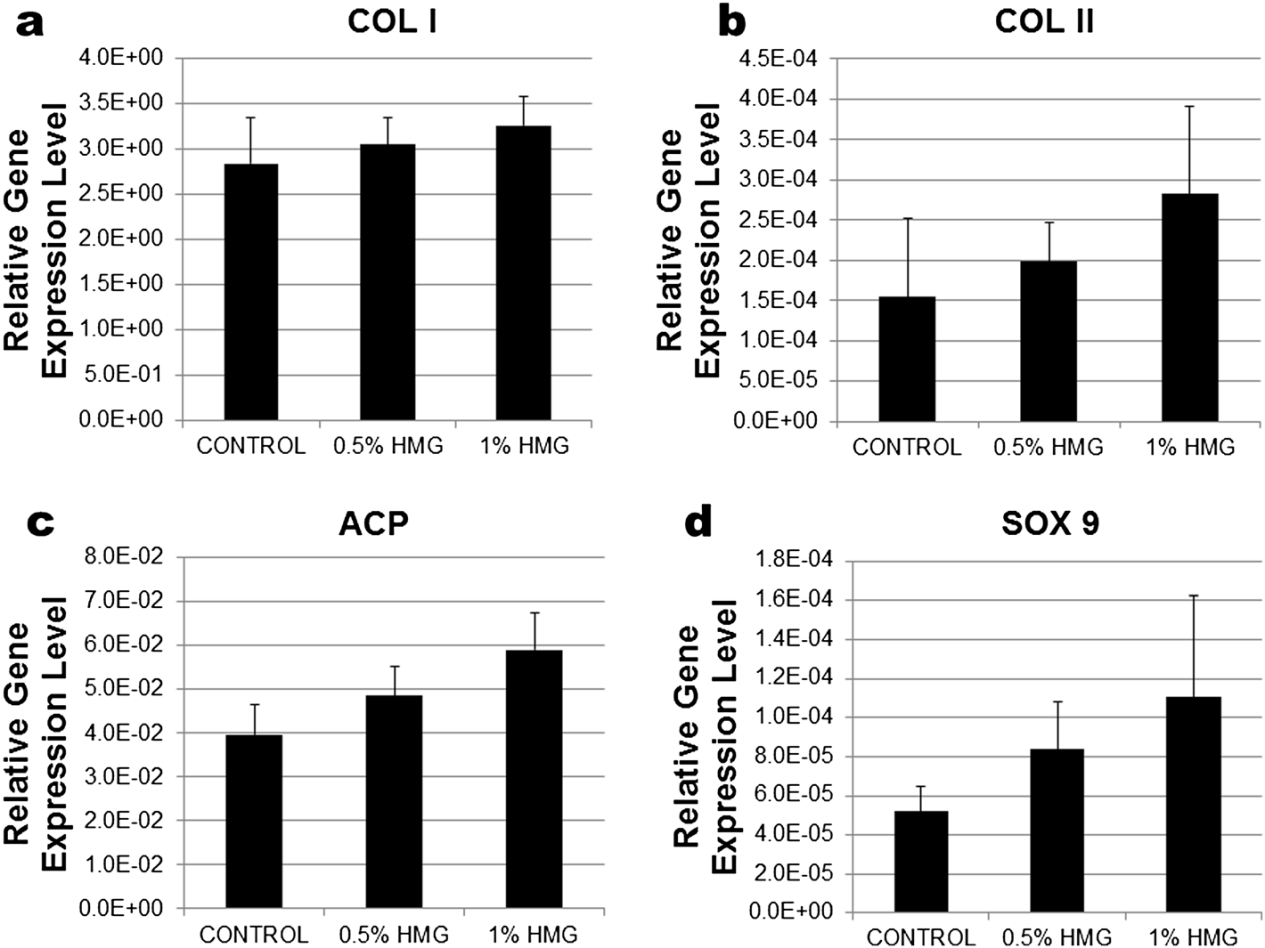
**Relative gene expression level of anabolic mediator in HACs cultured in medium supplemented with HMG. 6a**) Type I collagen (COL 1), **6b**) Type II collagen (COL II), **6c**) ACP and 6d) SOX-9. The values were expressed as mean ± SEM, n=6.

HMG supplementation increased the anabolic gene expression of cultured HACs. The expression of COL I, COL II, ACP and SOX-9 mRNA was increased in a dose-dependent manner of HMG supplementation (Figure 6). COL I expression was increased 1.15fold; COL II at +1.28fold; ACP at +1.49fold and SOX-9 at +2.13fold with 1.00% HMG supplementation. The chondrocytes phenotype index (ratio of COL II/COL I) also increased in a dose-dependent manner with HMG supplementation. The ratio of COL II/ COL I gradually increased from 1.19fold in 0.5% HMG to 1.33fold in 1.00% HMG supplementation (Table 2).

**Table 2:** Ratio of COL II/COL I in HACs cultured in medium supplemented with HMG

**HMG Concentration**	**COL II/COL I Index Relative to Control**
Control	1
0.5% HMG	1.195
1.0% HMG	1.33

### Prostaglandin E_2_ production in HACs

**Figure 7:**
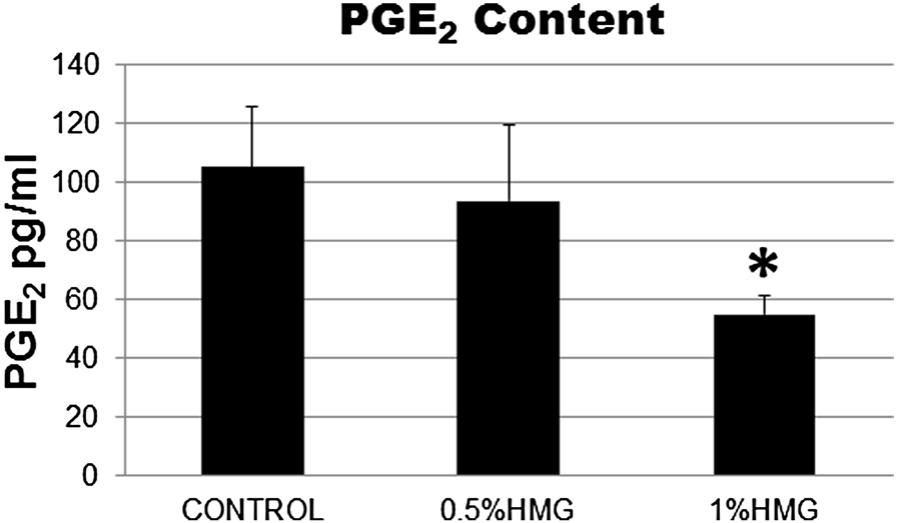
**Amount of PGE**_**2**_**produce in HACs cultured in medium supplemented with HMG.** The values were expressed as mean ± SEM, n=6. *Significant lower PGE_2_ compared to the control group (P<0.05).

The amount of PGE_2_ produced in HACs was decreased by −1.12fold in 0.50% HMG supplementation. The higher concentration of HMG at 1.00% caused a significant decrease in PGE_2_ production by −1.92fold compared to the control group (P=0.025; Figure 7).

### Sulphated glycosaminoglycan (sGAG) production in HACs

**Figure 8:**
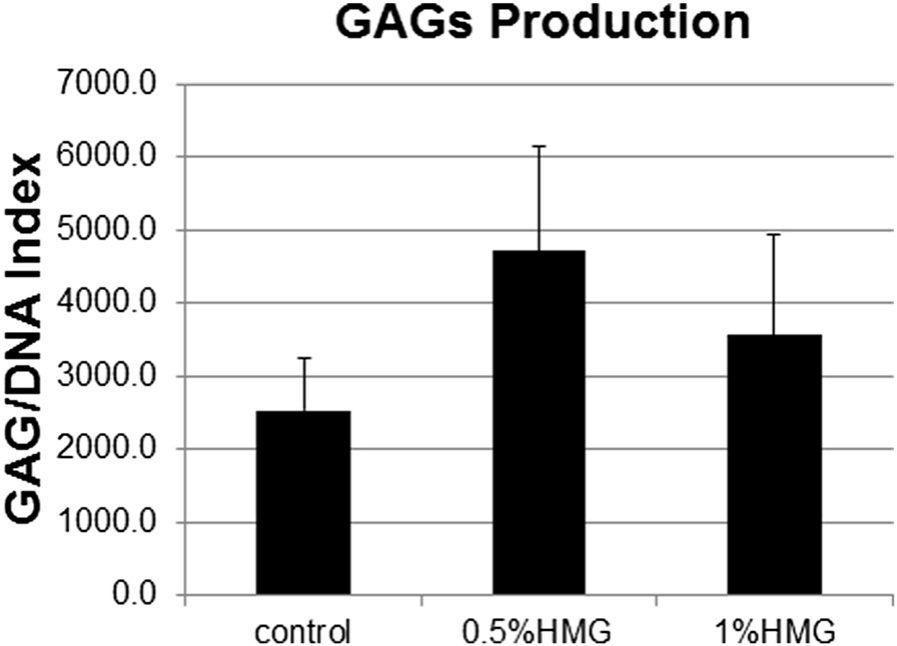
**Amount of GAGs production in HACs cultured in medium supplemented with HMG.** The values were expressed as mean ± SEM, n=6.

The amount of sGAG produced by the HACs in the control group was 2539.83±705.67 sGAG/DNA. Supple-mentation of HMG into the culture medium caused an increase in the production of sGAG to 4718.46±1421.08 sGAG/DNA (0.50% HMG) and 3572.58±1357.89 sGAG/DNA in 1.00% HMG (Figure 8).

## Discussion

The results of the present study showed EBN extract coded HMG worked effectively in low concentration on the HAC cultures. HMG was able to reduce the dendritic changes on HACs due to culture in serum-free medium. A previous study showed cultured chondrocytes changes its morphology when dedifferentiated in monolayer culture [19]. Only cultures with round-polygonal chondrocytes could synthesize type II collagen and maintain the differentiated phenotype [20]. Thus, HMG was able to reduce the dendritic changes and dedifferentiation on cultured HACs compared to the control group.

Matrix metalloproteinases (MMPs) are zinc-endopeptidases involved in numerous physiological and pathological events involving cartilage including degradation of both the endogenous and newly synthesized extracellular matrix proteins [21,22]. HMG supplementation may significantly decrease the expression of MMP1 and MMP3 in HACs compared to the control group. This helps in reducing the continuous destruction of cartilage and the degenerative progression of osteoarthritic cartilage [18,23]. The expression of pro-inflammatory cytokines (IL-1, IL-6 and IL-8) in cultured HACs supplemented with HMG was lower compared to the control group. A significant decrease in IL-1 was observed in the HMG supplemented group. IL-1 interacts with various cytokines including IL-6 and IL-8 [24] to induce the secretion of MMPs and reactive oxygen species that destroy the cartilage matrix [25]. The capability of HMG to suppress these cytokine’s expression may further decrease the MMPs activity and help in the reduction of the cartilage destruction [22].

In response to IL-1, chondrocytes increase the synthesis of nitric oxide (NO) and prostaglandin E_2_ (PGE_2_) through the activation of iNOS and cyclooxygenase-2 (COX-2) [26-28]. In this study, COX-2 and iNOS expression was reduced in HACs supplemented with HMG. Furthermore, PGE_2_ production was significantly decreased in HMG supplemented HAC cultures compared to the control group. The similar results were demonstrated in a previous study which used avocado soybeans extract [29]. Thus, the ability of HMG to decrease PGE_2_ production and down-regulate iNOS and COX-2 expression indicated its potential use in slowing down the cartilage destruction in OA.

In chondrocyte monolayer culture, dedifferentiation started when type II collagen expression reduced together with an increase of type I collagen (COL I) expression [30]. In this study, the expression of COL I and COL II mRNA did not state significant differences between the HMG supplemented groups compared to the control group. However, the ratio of COL II/COL I was increased with the supplementation of HMG. The increase of COL II/COL I ratio is necessary to reduce the chondrocytes’ dedifferentiation and improve the chondrocytes’ phenotype [31]. Besides, HMG supplementation also caused a gradual increase in SOX-9 and aggrecan core protein (ACP) expression in cultured HACs compared to the control group. SOX-9 is a transcription factor in chondrogenesis, and its expression is essential for the anabolic activity of the chondrocyte as well as the differentiation of stem cells [32]. While ACP is a major component of the cartilage matrix which helps the cartilage in sustaining continuous mechanical loads [33]. In the present study, both the ACP mRNA expression and sulphated glycosaminoglycans (GAGs) production were increased by HMG supplementation although no significant difference was recorded. Overall, HMG supplementation may need a longer duration of action before it can cause a significant increase in the anabolic activity of the cultured HACs.

## Conclusion

The catabolic activity of cultured chondrocyte was significantly reduced with the HMG supplementation. The ability of HMG to down-regulate MMPs, cytokines and other catabolic mediator expression suggest that HMG may be a potential anti-inflammatory/anti-degenerative agent in treating osteoarthritis. HMG also causes an increase in the chondrocytes’ anabolic activity, although longer duration may be needed to demonstrate the significant changes. Thus, it is concluded that HMG is a potential chondro-protective agent.

## Competing interests

The authors declare that they have no competing interest.

## Authors’ contributions

KHC had involved in the design of the study, coordination the experiment and written the manuscript. KN had carried out the study, performed the statistical analysis and drafted the manuscript. NHMY had provided the samples for the study and helped to draft the manuscript. LCT had participated in the sequence alignment, revised the manuscript. ETTT had participated in the design of the study, helped to draft the manuscript. LTH conceived the study and participated in the design and coordination. RAA helped to draft and revise the manuscript. All authors read and approved the final manuscript.

## Pre-publication history

The pre-publication history for this paper can be accessed here:

http://www.biomedcentral.com/1472-6882/13/19/prepub
